# Thy-1 (CD90), Integrins and Syndecan 4 are Key Regulators of Skin Wound Healing

**DOI:** 10.3389/fcell.2022.810474

**Published:** 2022-02-03

**Authors:** Leonardo A. Pérez, Lisette Leyton, Alejandra Valdivia

**Affiliations:** ^1^ Cellular Communication Laboratory, Program of Cellular & Molecular Biology, Center for Studies on Exercise, Metabolism and Cancer (CEMC), Instituto de Ciencias Biomédicas, Facultad de Medicina, Universidad de Chile, Santiago, Chile; ^2^ Advanced Center for Chronic Diseases (ACCDiS), Faculty of Chemical and Pharmaceutical Sciences, Universidad de Chile, Santiago, Chile; ^3^ Faculty of Medicine, Universidad de Chile, Santiago, Chile; ^4^ Division of Cardiology, Department of Medicine, Emory University, Atlanta, GA, United States

**Keywords:** Thy-1, CD90, integrins, mesenchymal cells, wound healing, cell migration, angiogenesis, syndecan 4

## Abstract

Acute skin wound healing is a multistage process consisting of a plethora of tightly regulated signaling events in specialized cells. The Thy-1 (CD90) glycoprotein interacts with integrins and the heparan sulfate proteoglycan syndecan 4, generating a trimolecular complex that triggers bi-directional signaling to regulate diverse aspects of the wound healing process. These proteins can act either as ligands or receptors, and they are critical for the successful progression of wound healing. The expression of Thy-1, integrins, and syndecan 4 is controlled during the healing process, and the lack of expression of any of these proteins results in delayed wound healing. Here, we review and discuss the roles and regulatory events along the stages of wound healing that support the relevance of Thy-1, integrins, and syndecan 4 as crucial regulators of skin wound healing.

## 1 Introduction

The skin is considered the largest human organ and functions as a natural barrier that protects the organs from environmental factors, such as light, heat, chemicals, dehydration, and infections (Yousef et al., 2020). After an acute skin injury, the body initiates a wound repair process, which is necessary to restore skin integrity and homeostasis. Wound repair consists of a plethora of tightly regulated biological and molecular processes that can be divided into four continuous and overlapping phases: hemostasis, inflammatory, proliferative, and remodeling. Hemostasis occurs immediately after an injury. Platelets aggregate and form a blood clot, which is mainly constituted by extracellular matrix (ECM) proteins forming the provisional matrix, which acts as a scaffold for cell migration (Chen and Lopez, 2005). The inflammatory phase involves the migration of cells, such as phagocytic neutrophils, macrophages, and leukocytes to the wound site. Subsequently, the phagocytic cells release cytokines and other soluble factors to induce fibroblast migration and proliferation (Martin and Leibovich, 2005; Nathan, 2006). During the proliferative phase, new blood vessels are formed (either by angiogenesis or neovascularization), giving rise to the synthesis of ECM components, as well as re-epithelialization. The final phase comprises collagen deposition and remodeling (Thiruvoth et al., 2015; Cañedo-Dorantes and Cañedo-Ayala, 2019).

These four phases can be arrested at any point, leading to the formation of a chronic non-healing wound. Alterations in any mediators, including soluble factors (e.g., inflammatory molecules and growth factors), proteases (e.g., matrix metalloproteinases), blood elements, the ECM, parenchymal and inflammatory cells, can also lead to impaired healing. Other comorbidities, such as diabetes, immunosuppression, renal failure, infection, and smoking, negatively affect the wound healing process.

Thy-1 (CD90), a glycosylphosphatidylinositol (GPI)-anchored protein, has been described as one of the cell receptors that participates in the acute wound healing process (Lee et al., 2013). Thy-1 is expressed in a variety of cells, including fibroblasts, neurons, endothelial, and hematopoietic cells. Thy-1 expression is tightly regulated during development, inflammation, and fibrosis. Moreover, Thy-1 is considered a cell marker for fibroblast and mesenchymal stem cells (MSCs), both of which play a transcendental role during wound repair. Most importantly, Thy-1 is the ligand/receptor for integrins and syndecan 4, and their interaction generates a trimolecular complex that triggers bi-directional signaling pathways to regulate several cellular processes, including cell adhesion, differentiation, migration, and proliferation (reviewed in Leyton et al., 2019).

Here, we review the direct role of Thy-1 and its co-receptors, integrins and syndecan 4, during the different phases of the acute skin wound healing process. Moreover, based on previously published functions related to other pathophysiological situations, we propose and critically discuss some new putative roles for the Thy-1/integrin/syndecan 4 trimolecular complex in the skin healing process, which could translate into potential therapeutics to improve clinical outcomes.

## 2 Thy-1, Integrins and Syndecan 4 Are Cell-Cell and Cell-Matrix Communication Molecules

Thy-1 is a small (25–37 kDa) glycoprotein that possesses two N-glycosylation sites in humans, and three in mice. Reports indicate differential Thy-1 expression between tissues and during development. In adults, Thy-1 protein is highly expressed in the brain, smooth muscle, kidney, and colon. In contrast, RNA studies have shown that Thy-1 is transcribed in many other tissues and cell types, such as the endometrium, adipose tissue, the urinary bladder, and T cells (Thul et al., 2017). During development, Thy-1 is undetectable in the neonatal and developing brain, compared to the higher levels observed in the adult brain. Thy-1 expression can also vary within the same cell type, defining cell subpopulations that possess different functions. For example, lung fibroblasts expressing higher levels of Thy-1 secrete ECM and inflammatory molecules different from those of fibroblasts expressing lower levels. Thy-1 presence in fibroblasts can also dictate if they differentiate into myofibroblasts or lipofibroblasts (Koumas et al., 2003).

Thy-1 localizes in lipid rafts at the cell membrane, but it can also be shed by specific phospholipases (PI-PLC or PLC-β) and other proteases not yet identified. Soluble Thy-1 (sThy-1) has been detected in serum, urine, wound fluid, and synovial fluid. *In vitro*, lung fibroblasts treated with IL-1β or TNFα shed Thy-1 into the culture media. Increased levels of sThy-1 exist in wound fluid from venous ulcers and synovial fluid from knee punction in patients with rheumatoid arthritis, as well as in serum from patients with systemic sclerosis and diabetic kidney disease, suggesting that sThy-1 exerts a role during inflammation and some pathological conditions (Freimuth et al., 1978; Saalbach et al., 1999; Kollert et al., 2013; Wu et al., 2021).

Thy-1 is a very versatile glycoprotein that can act as a receptor, a ligand, or a cell adhesion molecule. Thy-1 possesses an integrin-binding domain (RGD-like tripeptide: RLD) and a heparin-binding domain (HBD: REKRK, in mouse), which allow its interactions with several integrins and syndecan 4, respectively. The interaction of Thy-1 with its receptors occurs within the same cell (in *Cis*) or between cells (in *Trans*) to trigger diverse signaling pathways downstream of Thy-1, integrins, and/or syndecan 4 (reviewed in Herrera-Molina et al., 2013; Leyton et al., 2019).

Integrins are transmembrane receptors formed by α and β heterodimers that bind ECM proteins, cell surface molecules, and soluble ligands. At least 18 α and 8 β subunits have been described in humans, generating 24 different heterodimers, which recognize and interact with specific ligands. Integrins transduce signals from the ECM into the cell (outside-in), but the cell can also regulate the integrin affinity for its ligand (inside-out). To date, Thy-1 has been shown to interact with the αvβ3, αxβ2, αMβ2, α5β1, and αvβ5 integrins (Table 1) (Leyton et al., 2001; Wetzel et al., 2004; Saalbach et al., 2005; Hermosilla et al., 2008; Avalos et al., 2009; Zhou et al., 2010; Fiore et al., 2014).

**TABLE 1:** Thy-1 and its integrins/syndecan 4 receptors are expressed in different cell types that participate in the wound healing process.

Cell type	Integrins/syndecan 4 expression	Thy-1 expression	Effect
Platelets	αIIβ3^**a**^, α2β1^**a**^ (Rodrigues et al., 2019)	ND	May bind to endothelial cells expressing Thy-1
	αvβ1^**a**^, α6β1^**a**^, αvβ3, αIIbβ3^**a**^ (Bennett, 2005)		
	syndecan 4^**b**^ (Kaneider et al., 2005)		
Mast cells	α4^**a**^, α5^**a**^, α6^**a**^, β1, β7^**a**^ (Grodzki et al., 2003)	Thy-1 (Draberova et al., 1996)	Cell adhesion, stabilization of lipid rafts
	syndecan 4^**b**^ (Higashi et al., 2018)		
Monocytes	αMβ2 syndecan 4^**b**^ (Kaneider et al., 2002)	ND	Adhesion and migration
Macrophages	αMβ2	Thy-1 (Thul et al., 2017)	Adhesion and migration
	syndecan 4^**b**^ (Hamon et al., 2004)		
Leukocytes	αMβ2 (Wetzel et al., 2004)	ND	Extravasation of leukocytes Secretion of MMP9 and CXCL8
	syndecan 4^**b**^ (Gopal, 2020)		
MSCs	α3^**a**^_,_ αv (Lee H. M et al., 2020)	Thy-1 (Saalbach and Anderegg, 2019)	Cell differentiation
Keratinocytes	αvβ5^**a**^, αvβ6^**a**^, α5β1^**a**^,	Thy-1 (Nakamura et al., 2006)	Migration and cell proliferation
	α3β1^**a**^, α6β4^**a**^		
	syndecan 4^**b**^ (Rousselle et al., 2019)		
Endothelial cells	β3^**b**^ (Sepp et al., 1994)	Thy-1^**b**^ (Lee et al., 1998)	Transendothelial migration of leukocytes
	syndecan 4^**b**^ (Vuong et al., 2015)		
Pericytes	α6β1^**a**^ (Reynolds et al., 2017)	Thy-1 (Billaud et al., 2017)	Angiogenesis and deposition of the ECM
	syndecan 4 ND		
Fibroblasts	αvβ3, αvβ1 (Bates et al., 1991)_,_ α5β1	Thy-1 (Koumas et al., 2003)	Cell differentiation, latent activation of TGFβ; expression of PDGF, the ECM and cytokines
	syndecan 4^**b**^ (Fiore et al., 2014)		
Schwann	αvβ3 (Milner et al., 1997), α5β1^**b**^ (Lefcort et al., 1992)	ND	May bind to endothelial cells expressing Thy-1
	syndecan 4^**b**^ (Goutebroze et al., 2003)		

ND, not determined.

a Integrin heterodimers or monomers have not been described as a Thy-1 ligand.

b Expression is either triggered or increased after injury or inflammation.

The syndecan family is comprised of four members (syndecan 1, 2, 3, and 4), which are timely and spatially expressed across every cell of the body. Syndecans are transmembrane heparan sulfate proteoglycans, composed of a divergent ectodomain, a conserved transmembrane region, and a short cytoplasmic tail. The ectodomain possesses glycosaminoglycan chains (GAGs) of heparan sulfate that interact with ECM proteins, cytokines, chemokines, growth factors, and its receptors. The ectodomain can also be shed from the cell surface to sequester soluble factors or compete for binding to the ECM. The cytoplasmic domain possesses two conserved regions (C1 and C2), flanking a variable (V) region unique to each syndecan, which triggers specific cell signaling pathways (Simons and Horowitz, 2001; Bertrand and Bollmann, 2019; Gondelaud and Ricard-Blum, 2019).

Of interest for this review, integrins act cooperatively with syndecan 4 to regulate focal adhesion (FA) and actin stress fiber formation in a RhoA-dependent manner (Avalos et al., 2009; Fiore et al., 2014). FA turnover is an important event during directional cell migration. Moreover, Thy-1 has been shown to directly interact with integrins and syndecan 4 to form a trimolecular complex that facilitates initial cell adhesion by enhancing FA formation and subsequently, promoting migration by regulating contractility and FA turnover (Avalos et al., 2004; Hermosilla et al., 2008; Avalos et al., 2009; Kong et al., 2013; Fiore et al., 2014; Fiore et al., 2015; Lagos-Cabre et al., 2017; Burgos-Bravo et al., 2020; Valdivia et al., 2020). Although the bidirectional signaling pathways triggered by Thy-1/integrin/syndecan 4 clustering and activation remain unexplored during skin injury, the biological significance of each of these molecules by themselves has been proven important for an efficient wound healing process.

Most of the cell types participating in the wound healing process express integrins, syndecan 4 and Thy-1 (Table 1). Furthermore, Thy-1, integrins and syndecan 4 are upregulated after skin injury and in response to inflammation (Table 1) (Lefcort et al., 1992; Sepp et al., 1994; Gallo et al., 1996; Lee et al., 1998; Goutebroze et al., 2003). The Thy-1 promoter becomes active right at the wound area and remains active up to 36 days after injury (Josvay et al., 2014). Similarly, transcriptome analysis has shown that human blood vessels in a wounded area express 24-fold higher Thy-1 levels than normal tissue vessels (Roy et al., 2007). Additionally, decreasing Thy-1 levels delays the wound healing process by generating abnormal re-epithelialization and TGFβ secretion (Lee et al., 2013).

Syndecan 4 is also upregulated in the epidermis after injury, and disruption of the syndecan 4 gene in mice delayed skin wound healing and impaired angiogenesis (Gallo et al., 1996; Echtermeyer et al., 2001). Syndecan 4 regulates wound healing *in vitro* by controlling the levels of integrins at the membrane to allow an efficient directional cell migration, and *in vivo*, by upregulating its levels within the granulation tissue, implying a role during wound-related angiogenesis (Fuster and Wang, 2010; Bass et al., 2011; Brooks et al., 2012; Vuong et al., 2015).

On the other hand, integrins have been proven transcendental for the recruitment of leukocytes, keratinocytes, and fibroblasts to the wound area, as well as for myofibroblast differentiation and blood vessel sprouting during angiogenesis (Columbo and Bochner, 2001; Annes et al., 2004; Asano et al., 2005; Asano et al., 2006; Carracedo et al., 2010; Zarbock et al., 2012; Darby et al., 2014; Koivisto et al., 2014; Kenny and Connelly, 2015; DiPersio et al., 2016; Lishko et al., 2018). Aberrant integrin signaling is associated with defective ECM deposition, which affects normal fibroblast differentiation and function, and also generates inefficient cell recruitment and insufficient angiogenesis, which cause a hypertrophic scar or chronic wounds (Koivisto et al., 2014).

Substantial evidence supports the importance of Thy-1 and its counteracting receptors during wound closure. In the next sections, we review the specific roles that Thy-1, integrins, and syndecan 4 play in each one of the phases of the acute skin wound healing process, with a special emphasis on yet unexplored functions, which may be relevant for future therapeutic development.

## 3 Hemostasis Phase

The immediate response after an acute wound is vasoconstriction and clot formation to prevent blood loss. Vasoconstriction is mediated by prostaglandins and endothelin released from circulating platelets and the damaged endothelial layer (Menter et al., 2017; Rodrigues et al., 2019). Moreover, circulating catecholamines and prostanoids, such as epinephrine, norepinephrine, and thrombocyclin can also generate vasoconstriction (Feletou et al., 2010). Platelet-derived growth factor (PDGF) activates smooth muscle cells in the vessel to cause contraction (Berk et al., 1986). After the initiation of the coagulation cascade, vasoconstriction resolves the bleeding through thromboxane A2, bradykinin, serotonin, and fibrinopeptide (Rodrigues et al., 2019). The clot formation process is initiated in response to the vascular wall damage, which exposes the subendothelial collagen to blood components (Chen and Lopez, 2005). Platelets adhere to collagen, become activated, and aggregate to form the initial hemostatic plug. At this point, the coagulation and complement cascades are activated, mediating the cleavage of prothrombin to thrombin, which subsequently cleaves fibrinogen to fibrin. Fibrin strands bind at the hemostatic plug along with platelets and erythrocytes to form an insoluble clot. Apart from the crosslinked fibrin strands, the clot also contains other ECM proteins, such as collagen type I, fibronectin, vitronectin, and thrombospondin (TSP), which create a provisional ECM that favors fibroblast and leukocyte migration. The active platelets in the clot will also undergo degranulation, and release soluble factors to mediate vasoconstriction, endothelial and fibroblast activators (e.g., TGFβ, VEGF, FGF2, and PDGF), and chemoattractant and inflammatory mediators [e.g., chemokine (C-X-C motif) ligand 4 (CXCL4 or PF4), chemokine (C-C motif) ligand 5 (CCL5), Interleukin 6 (IL-6), Interleukin 8 (IL-8), and Insulin growth factor 1 (IGF1)] (Martins-Green et al., 2013; Ridiandries et al., 2018; Cañedo-Dorantes and Cañedo-Ayala, 2019; Rodrigues et al., 2019).

Integrin αIIbβ3 is the main integrin on the surface of platelets and mediates adhesion to fibrinogen, fibronectin, vitronectin, and the Von Willebrand Factor (vWF). The function of αIIbβ3 is indispensable for platelet aggregation, clots retraction, and thrombus stability during wound healing. Platelets also express, to a lesser extent, α2β1, α5β1, α6β1, and αvβ3 integrins (Table 1) (Bennett, 2005; Bennett et al., 2009; Haling et al., 2011; Eisinger et al., 2018). Although, α2β1 mediates adhesion to collagen and αvβ3 to osteopontin, as well as to vitronectin during *in vitro* assays, it is uncertain whether they play a role during hemostasis or if they can interact with Thy-1 expressed on other cell types present in the wounded area (Bennett et al., 1997; Paul et al., 2003).

Syndecan 4 is ubiquitously expressed in adult human cells. Particularly in healthy skin, tissue analysis shows medium-to-high levels of syndecan 4 protein expression in keratinocytes, Langerhans (tissue-resident macrophages in epidermis), fibroblasts, and epidermal cells. Single-cell RNA analysis has also shown that skin endothelial cells, smooth muscle cells, melanocytes and T cells express syndecan 4 (Thul et al., 2017; Sole-Boldo et al., 2020; Karlsson et al., 2021; The Human Protein Atlas, 2021). Platelets themselves express syndecan 4, and clustering of syndecan 4 using antibodies increases platelet aggregation, though the role of syndecan 4 in platelet aggregation has not been described during skin hemostasis (Kaneider et al., 2005).

**FIGURE 1:**
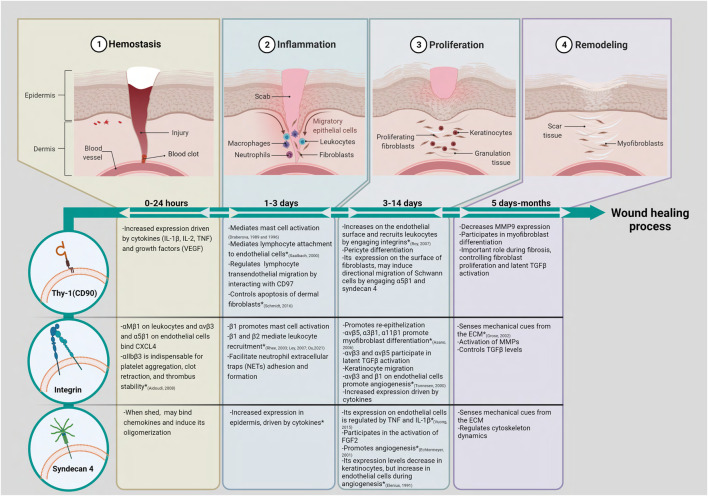
Thy-1 (CD90), integrins, and syndecan 4 could participate in multiple stages of skin wound healing. Based on data obtained in similar cell types from organs other than skin, we have proposed putative roles for Thy-1, integrins and syndecan 4 during the wound healing process. (*) Shows functions for these molecules that have already been studied in skin and/or skin wound models. In brief, the acute skin wound healing process consists of four phases that overlap and are tightly regulated. (1) The hemostasis phase starts immediately after injury. Platelets aggregate and form a blood clot containing ECM proteins and secrete soluble factors, such as CXCL4, which lead cell migration into the wound area; (2) The inflammatory phase involves the migration of neutrophils, macrophages, and leukocytes to the wound site. The inflammatory cells release cytokines, chemokines, and growth factors to recruit fibroblasts into the wound; (3) The proliferation phase is characterized by proliferation of fibroblasts, angiogenesis, formation of granulation tissue, peripheral nerve repair, recruitment of keratinocytes, and re-epithelization; (4) The remodeling phase is characterized by wound contraction and collagen remodeling. The roles for Thy-1 (CD90) and its co-receptors integrins and syndecan 4 are highlighted in each phase. Created with Biorender.com.

Single-cell RNA analysis of healthy skin shows that Thy-1 is normally expressed in fibroblasts and smooth muscle cells, while lower levels are observed in endothelial cells, keratinocytes, melanocytes, resident skin macrophages, and granulocytes (Thul et al., 2017; Sole-Boldo et al., 2020; Karlsson et al., 2021; The Human Protein Atlas, 2021). Even though Thy-1 is considered a dermal fibroblast and dermal mesenchymal stem cell marker, its expression levels in healthy skin are very low compared to other tissues, such as the cerebral cortex (<30-fold) (Thul et al., 2017; Sole-Boldo et al., 2020; Karlsson et al., 2021; The Human Protein Atlas, 2021). To date, it remains unknown whether Thy-1 basal levels play a relevant role in skin physiology or during the hemostasis phase. However, Thy-1 levels rapidly increase after skin injury (Figure 1). Indeed, using an ear injury model on transgenic mice expressing YFP under the control of the Thy-1 promoter, Jósvay et al., showed that a halo of fluorescence appears right at the wound edge, as soon as day 1 after injury. At day 3, the fluorescent area expands to the surrounding wounded area, and the fluorescence remains up to 2 weeks after the injury (Josvay et al., 2014). The fluorescent area around the wound starts decreasing at day 21 and disappears by day 36, indicating that the activity of the Thy-1 promoter is tightly controlled during the wound healing process (Freimuth et al., 1978). These changes in Thy-1 expression presumably occur in response to the inflammatory molecules and growth factors that are released in the wound area early during the hemostasis phase. Supporting this idea, cytokines such as IL-1β, IL-2, and TNFα, and growth factors such as VEGF can induce Thy-1 synthesis in other systems (Mason et al., 1996; Lee et al., 1998; Weston et al., 2002; Zhao et al., 2003; Wetzel et al., 2004). Notably, IL-1β, IL-6, and TNFα levels are elevated in the diabetic wound healing process. High levels of these chemokines during hemostasis activates the secretion of acute-phase proteins from the liver and recruit an excessive number of inflammatory cells to the wound site, thus affecting the healing process (Strang et al., 2020). Moreover, Thy-1 levels are also increased during diabetic foot ulcers (Januszyk et al., 2020). Therefore, we speculate that at least during diabetic wound healing these cytokines can induce Thy-1 overexpression to mediate the migration of inflammatory cells. Some of the mechanisms by which Thy-1 can stimulate cell migration on the surface of inflammatory cells will be discussed in detail in the description of the inflammatory phase (Section 4). Similarly, the expression of integrins and syndecan 4 is also regulated by inflammatory mediators (Herzberg et al., 1996; Werner and Grose, 2003; Okuyama et al., 2013; Lagos-Cabre et al., 2017; Schnittert et al., 2018; Gopal, 2020). It is noteworthy that CXCL4, the main chemokine secreted by platelets during the hemostasis phase, binds integrin αMβ2 (MAC-1) on leukocytes, as well as αvβ3 and α5β1 on endothelial cells (Figure 1) (Aidoudi et al., 2008; Lishko et al., 2018). Therefore, Thy-1 could potentially modulate the effects of CXCL4 by competing for its integrin receptor, although this idea needs to be evaluated in the context of skin wound healing.

The role of syndecans in the activation of chemokines has been broadly studied. Chemokines such as CCL5 and CXCL4, which are secreted by activated platelets during the hemostasis phase, require both chemokine oligomerization and binding to GAGs to participate in cell recruitment. CCL5 induces syndecan 4 shedding from the surface of HeLa cells, and syndecan 4 can bind chemokines and induce chemokine oligomerization (Figure 1) (Charnaux et al., 2005; Dyer et al., 2016). Nonetheless, further research is necessary to establish a specific role of syndecan 4 in the oligomerization of chemokines during the hemostasis phase after acute skin injury.

Thy-1 can also be related to the secretion of ECM in other cell types not associated with skin. For instance, the induction of Thy-1 in human ovarian cancer cells enhances the expression of fibronectin and TSP1 (Figure 1). In addition, pulmonary Thy-1 (+) and Thy-1 (−) fibroblasts synthesize fibronectin, but the subpopulation of Thy-1 (+) fibroblasts produces two- to three-fold more collagen than the Thy-1 (−) cells (Derdak et al., 1992; Abeysinghe et al., 2005). Moreover, TSP1, one of the provisional ECM components, induces the disassembly of focal adhesions necessary to induce fibroblast migration in a Thy-1-dependent manner (Barker et al., 2004). However, it remains unknown whether Thy-1 can trigger ECM secretion or interact with TSP1 during skin wound healing.

Therefore, the expression of Thy-1, integrins, and syndecan 4 can potentially be modulated by soluble signals released during hemostasis, although more research is necessary to elucidate if Thy-1 and its receptors also exert a role in controlling key events for hemostasis, such as activation of chemokines, ECM secretion and early cell migration to the wounded area.

## 4 Inflammatory Phase

During the inflammatory phase, neutrophils, macrophages, and lymphocytes are recruited to the wound site. Bradykinin and anaphylatoxins (C3a and C5a), generated by the coagulation and complement cascades, respectively, disrupt cell-cell junctions of the endothelial cells and increase the permeability of the local vessels, facilitating the infiltration of inflammatory cells. Neutrophils are the first to arrive to the wound area, following a gradient of chemoattractant molecules composed of several growth factors and chemokines released by activated platelets in the blood clot, as well as N-formyl peptides released by bacteria and damaged cells (Mantovani et al., 2011). Within the wound, neutrophils will decontaminate the wound using diverse strategies, including phagocytosis, proteases, secretion of antimicrobial peptides, generation of reactive oxygen species (ROS), and neutrophil extracellular traps (NETs) to immobilize and kill microorganisms (Brinkmann et al., 2004; Nathan, 2006; Kolaczkowska and Kubes, 2013). Despite their relevant role in controlling infection, the absence of neutrophils does not impair the healing process. Indeed, wound repair can happen faster in animals deficient in neutrophils, suggesting that neutrophils can be inhibitory to some extent during the repair process (Simpson and Ross, 1972; Dovi et al., 2003; Martin and Leibovich, 2005). On the other hand, the sustained presence of neutrophils in the wound could be one of the causes of chronic non-healing wounds (Martin and Leibovich, 2005).

In addition to NET function as a host defense mechanism, NETs also participate in thrombus formation and metastatic dissemination of cancer cells. In this context, α9β1 integrin in the neutrophil plasma membrane promotes thrombosis and clot formation, and the adhesion of different tumor cells to NETs is facilitated by high expression of α5β1, αvβ3, and αvβ5 integrins (Martinod and Wagner, 2014; Monti et al., 2018). Similarly, integrins reportedly regulate neutrophil activation and NET formation (NETosis). In a mouse model of ventilator-induced lung injury, blocking integrin-mediated outside-in signaling decreases NET formation and lung injury (Rossaint et al., 2014). Alternatively, stimulation of neutrophils with phorbol 12-myristate 13-acetate (PMA), a potent protein kinase C (PKC) activator, induces complete NET formation independent of cell adhesion (Erpenbeck et al., 2019). Therefore, NET formation could occur in an integrin-dependent and -independent manner. However, the exact mechanism by which PMA induces NET formation remains unclear. Currently, there is no evidence that the Thy-1/integrin/syndecan 4 trimolecular complex can control NETosis, although, syndecan 4 can regulate PKC activity, and PMA can increase Thy-1 expression on endothelial cells to facilitate wound healing (Oh et al., 1997; Murakami et al., 2002; Wen et al., 2018). More importantly, increased NETosis is associated with delayed wound healing in diabetic skin wounds, which therefore posit NET formation mechanisms as an attractive subject of study (Wong et al., 2015; Fadini et al., 2016; Erpenbeck et al., 2019; Lee, Y. S. et al., 2020).

Following neutrophils, monocytes are recruited to the wound via C-C chemokines, such as CCL2. These chemokines are secreted initially by neutrophils and subsequently by keratinocytes and monocytes themselves (Gillitzer and Goebeler, 2001; Rees et al., 2015; Ridiandries et al., 2018). Within the wound, monocytes mature into macrophages, which initially acquire a pro-inflammatory phenotype (M1), and subsequently, as the wound heals, they transition to an anti-inflammatory and pro-regenerative phenotype (M2). As proposed by Krzyszczyk and other researchers, the M1/M2 definition is oversimplified, since in the wound bed, macrophages exhibit a different spectrum of M1- or M2-like characteristics (Martinez and Gordon, 2014; Krzyszczyk et al., 2018). M1-like macrophages remove dead cells, apoptotic neutrophils, bacteria, tissue debris, and foreign materials. They function as antigen-presenting cells, and secrete cytokines and growth factors, such as TGFα, TGFβ, bFGF, VEGF, and PDGF (Martins-Green et al., 2013; Krzyszczyk et al., 2018). These soluble factors attract and activate endothelial cells, fibroblasts, and keratinocytes. Subsequently, M2-like macrophages favor wound healing by inducing cell proliferation, angiogenesis, and ECM synthesis during the proliferative phase, and also by secreting matrix metalloproteinases (MMPs) in the remodeling phase, as discussed later (Sections 5 and 6). In a guinea pig wound model, macrophage depletion using antisera and steroids results in impaired disposal of damaged tissue and matrix, a decreased fibroblast count, and delayed wound healing (Leibovich and Ross, 1975; Martin and Leibovich, 2005). Surprisingly, antisera depletion of neutrophils does not affect the healing process (Simpson and Ross, 1972). More recent research using leukocyte-deficient mice has shown that not one of the inflammatory cell lineages is absolutely necessary to favor wound healing. Indeed, the absence of some leukocytes lineages can cause a faster wound repair and can also diminish scarring later during remodeling phase, suggesting that inflammatory cells can have an inhibitory effect during healing. In this context, neutrophil knockdown mice show faster wound repair than the control littermates, if the conditions are sterile (Dovi et al., 2003). Additionally, in PU.1 null mice, which lack macrophages and neutrophils, wound healing is not impaired; it follows a similar time course as in their WT littermates and healing occurs in the absence of fibrosis and scar formation, similarly to embryonic wound healing (Martin et al., 2003). Cytokines and growth factor levels are reduced in PU.1 null mice. However, they are not entirely absent, as observed in wounded embryonic tissue, since these soluble signals can still be produced in small amounts by keratinocytes and fibroblasts, suggesting that inflammation and macrophages are somehow not crucial for healing, although they may play an essential role in scar formation.

Evidence suggests that Thy-1 might mediate the recruitment of inflammatory cells to the wound site. As reported, Thy-1 expressed on endothelial cells mediates the binding of neutrophils and monocytes to activated microvascular endothelial cells (Figure 1) (Saalbach et al., 2000). Furthermore, αMβ2 integrin (Mac-1 or CD11b/CD18) expressed in the leukocyte membrane was identified as the counterreceptor for Thy-1 (Wetzel et al., 2004). Moreover, Thy-1 mediates the extravasation of monocytes and neutrophils in a thioglycollate-induced peritonitis model and the extravasation of eosinophils and monocytes in a lung inflammation model (Schubert et al., 2011). Additionally, the interaction between Thy-1 and αMβ2 integrin on neutrophils triggers effector functions in neutrophils, inducing the secretion of MMP9 and CXCL8 (Saalbach et al., 2008). Hence, by bringing together inflammatory cells to the wound bed, Thy-1 may contribute to modulate the inflammatory microenvironment.

The innate skin immune system, including neutrophils and monocyte/macrophages, provides a non-specific first-line response to pathogens, toxins, and foreign material. The innate response comprises toll-like receptors (TLRs) and receptor for advanced glycation end products (RAGE) receptors that recognize stress signals, such as lipopolysaccharide (LPS). These receptors then trigger signal transduction pathways that culminate with the release of TNF, IL-1β, IL-6, and NO. On the other hand, B- and T-lymphocytes complement the innate response more specifically: B-lymphocytes produce specific antibodies, and T-lymphocytes secrete cytokines and elicit cytolytic activity (Strbo et al., 2014). B-lymphocytes are present in the wound area from day 4 to day 17 after injury (Sirbulescu et al., 2017). In splenectomized nude mice, wound healing is delayed, and this effect is recovered after the addition of external antibody-producing B cells (Nishio et al., 2009). Similarly, topical treatment with B cells improves healing of acute wounds by 2–3 days in wild-type animals and 5–6 days in obese diabetic mice (Sirbulescu et al., 2017). Thy-1 is expressed on early B-cells in the thymus and has been related to the proliferation of B-cell lymphomas (Ritter et al., 1983; Ishiura et al., 2010). Nonetheless, Thy-1 is not expressed in mature B-cells. Relatedly, syndecan 4 blocking antibodies inhibit the directional migration of B-cells in an asthma model, and B-cells from syndecan 4^−/−^ mice also show impaired directional cell migration in an arthritis model (Endo et al., 2015; Polte et al., 2015). Although these results suggest a possible role of syndecan 4 during B-cell migration, further experiments are necessary to elucidate its role in skin wound repair.

T-lymphocytes in the skin consist of regulatory cells, CD4^+^ helper cells, and CD8^+^ killer cells, which can be present in the circulation or be permanent residents of the skin. Regulatory CD4^+^ T cells migrate and accumulate in the skin, where they participate in skin homeostasis and tolerance to normal skin flora (Ali and Rosenblum, 2017). The role of regulatory T cells in skin injury was recently reviewed (Boothby et al., 2020). In brief, regulatory T cells control inflammation and reduce the number of macrophages and upregulate the expression of EGFR to favor re-epithelialization and wound closure. As proposed, skin regulatory T cells play an important role in preventing an immune response against self-antigens during cutaneous injury (Metzger and Anderson, 2011). On the other hand, helper CD4^+^ T cells contribute to the inflammatory response against pathogens by releasing cytokines that mediate the secretion of antimicrobial peptides. Several subsets of helper T cells (Th1, Th2, Th17, and Th22) secrete unique cytokines, which orchestrate defensins and antimicrobial peptides to protect the skin from infection. Skin resident dendritic cells participate in the phagocytosis of microorganisms and present the antigens to naïve CD8^+^ cells. Active CD8^+^ killer cells differentiate in homing effector memory (T_EM_) T cells and central memory (T_CM_) T cells. T_EM_ cells migrate to the wound area and release proinflammatory cytokines to mediate pathogen clearance. After the infection is resolved, most of the T_EM_ cells die by apoptosis, and the few remaining cells are known as tissue-resident memory (T_RM_) T cells. After reinfection, dendritic cells present the antigen to the T_RM_ cells, which proliferate and recruit circulating T_EM_ cells to mediate pathogen clearance. Human hypertrophic scars generated after a burn injury show a high infiltration of T cells, and murine models have shown that scar formation is mediated by cytokines secreted by Th2 helper T cells (Cañedo-Dorantes and Cañedo-Ayala, 2019; Rodrigues et al., 2019). Interferon γ secreted by Th1 cells can attenuate tissue fibrosis by downregulating collagen synthesis and decreasing fibroblast proliferation (Harrop et al., 1995; Wynn, 2004). Similarly, keloid fibroblasts synthetize less collagen when co-cultured with regulatory T cells (Murao et al., 2014; Chen et al., 2019). Therefore, regulatory T cells maintain immune homeostasis.

Thy-1 can also induce T lymphocyte activation. Thy-1 is abundantly expressed on CD4^+^ CD8^+^ double-positive thymocytes and, to a lesser extent, at the T cell surface (Haeryfar and Hoskin, 2004). Although early research showed that Thy-1 expression was restricted to mature mouse T cells and that mature human T cells did not express Thy-1, more recent reports have shown that Thy-1 is expressed in specific subsets of human T cells (Th17/Tc17) (Guillot-Delost et al., 2012). Differentiation into Th17/Tc17 is enhanced after tissue damage and the dysregulation of these cells can lead to skin inflammatory conditions, such as atopic dermatitis, psoriasis, and Lichen Planus (Baurecht et al., 2015; Brockmann et al., 2017; Linehan et al., 2018; Norsgaard et al., 2019; Solimani et al., 2019). Moreover, single-cell RNA data has shown that T cells express low Thy-1 levels in healthy human skin; nonetheless, the significance of this observation has not been studied in healthy skin nor in wound healing models (Thul et al., 2017; Sole-Boldo et al., 2020; Vorstandlechner et al., 2020; Karlsson et al., 2021; The Human Protein Atlas, 2021). The role of Thy-1 in T cell function has been studied primarily on T cells from rodent models. In this context, it is known that T cells from Thy1^−/−^ mice show enhanced T cell antigen receptor (TCR) activity. Additionally, anti-Thy-1 antibodies activate mouse T cell proliferation and cytokine synthesis in the absence of an antigen-specific signal in the context of CD28 co-stimulation (Pont, 1987; Hueber et al., 1994) or a co-stimulatory signal from syngeneic bone marrow-derived dendritic cells (Haeryfar and Hoskin, 2001). Thus, Thy-1 can regulate the T cell response in the absence of TCR activation and enhance antigen-induced T cell responses in mouse cells. Noteworthy, Thy-1-regulated TCR activation has not been shown in human skin resident T cells during wound healing nor in the aforementioned skin conditions, where Th17/Tc17 cells are relevant.

The last leukocytes recruited to the wound area are the mast cells, which typically reside in the skin. They play an essential role in innate immunity during the healing process of infected wounds, where they release TNFα to chemoattract professional phagocytic cells, such as polymorphonuclear neutrophils (PMN). Knockout of mast cells in mice impairs wound healing and reduces the inflammatory response that diminishes fibrosis and scar formation (Szpaderska et al., 2003; Gallant-Behm et al., 2008; Shiota et al., 2010). In the activation of mast cells, Thy-1 membrane microdomains serve as a platform to aggregate Src-related proteins to induce activation of rat mast cells (Figure 1) (Draberova, 1989; Draberova et al., 1996). This activation also involves Thy-1 ligands, such as integrins, and in this context, the engagement of β1 integrin in mast cells increases their sensitivity to cellular activation (Ra et al., 1994). Mast cells can show an adherent or non-adherent behavior according to the integrin profile, which might include the expression of α4, α5, α6, β1, and β7 integrin subunits. The expression of α4 integrin is higher in adherent mast cells (Grodzki et al., 2003). β1 and αvβ3 integrin expression have also been related to mast cell adhesion in human skin (Columbo et al., 1995; Columbo and Bochner, 2001). Interestingly, α5β1 and αvβ3 integrins are reported Thy-1 receptors/ligands, and therefore, their interaction in this biological process could potentially contribute to the adhesive behavior of mast cells.

A role of Thy-1 in recruiting leukocyte cells and regulating inflammation has been sustained by studies that show that monocytes and neutrophils expressing αxβ2 and αMβ2 integrins use Thy-1 expressed on the surface of activated endothelial cells to migrate (Wetzel et al., 2004). The recruitment of leukocytes and the subsequent increase in cytokines can exacerbate the expression of Thy-1 in the endothelial cells at the wound site, as discussed during angiogenesis in the proliferative phase (Section 5). Thy-1 also interacts with CD97, an adhesion G-protein coupled receptor (GPCR) present at the adherent junctions formed between endothelial cells; such interaction probably mediates the transmigration of the leukocytes to the wound (Figure 1). Additionally, Thy-1 regulates the extravasation of leukocytes during acute lung inflammation, controlling the recruitment of different cells and preparing the inflammatory environment (Schubert et al., 2011).

Inflammatory cell recruitment and subsequent secretion of regenerative factors at the wound bed are tightly coordinated processes (Cañedo-Dorantes and Cañedo-Ayala, 2019). As discussed before, changes in the timing and inflammatory components can both positively and negatively affect the healing process. Another component reviewed in the literature is the heparan sulfate proteoglycan syndecan. Its role during inflammation depends on the degree of sulfation of the heparan sulfate chains, the rate of ectodomain shedding, the solubility of the ectodomains, and the expression levels of different syndecan family members (Gopal, 2020).

On the other hand, integrins mediate leukocyte accumulation at the sites of inflammation. Inflammatory mediators trigger integrin activation, and this is a crucial step to initiate leukocyte migration to inflamed tissues. Subsequently, integrin deactivation is indispensable for maintaining proper leukocyte migration (Zarbock et al., 2012; Park et al., 2015). Integrins are also important for leukocyte adhesion and transmigration through blood-vessel walls to get access to the site of inflammation, in neutrophil recruitment, lymphocyte recirculation, and monocyte trafficking. Specifically, β1 and β2 integrins have been addressed as central players in regulating leukocyte recruitment and vascular permeability during acute inflammation (Figure 1) (Rhee et al., 2003; Ley et al., 2007; Ou et al., 2021). Taken together, Thy-1 and its receptors integrins and syndecan 4 play a crucial role in recruiting leukocytes and restraining the immune response in the wound area. Therefore, further studies are necessary to evaluate if, for instance, the shedding of Thy-1 and syndecan 4 or changes in the expression of these receptors are important to clear up inflammation and lead to the remodeling phase of wound healing.

Lymphocyte-induced inflammation is cleared by a massive apoptosis process induced by interferon (INFc) and TNFα production at the wound site (Martins-Green et al., 2013). Liu et al., demonstrated that Bcl-2 and Bcl-xL synthesis was decreased in response to Thy-1 expression and could lead to apoptosis by activating the effector caspase-3 and poly ADP-ribose polymerase (PARP). In addition, they demonstrated that Thy-1 mediates apoptotic signaling via both caspase-9- and caspase-8-dependent pathways (Liu X. et al., 2017). Moreover, Thy-1/β3 integrin-induced apoptosis of dermal fibroblasts is mediated by upregulation of Fas ligand (FasL) expression (Figure 1) (Schmidt et al., 2016). FasL binds to and activates Fas, a cell-surface death receptor belonging to the TNF receptor superfamily, to induce apoptosis through the activation of caspase-8. Therefore, we hypothesize that Thy-1 and β3 integrin could play a vital role in terminating the inflammatory phase by inducing apoptosis of lymphocytes.

## 5 Proliferative Phase

During the proliferative phase, fibroblasts, keratinocytes, and endothelial cells divide, differentiate, and migrate to the wound area. This phase overlaps with the inflammatory phase, starts 2 days after injury with the degradation of the fibrin matrix and invasion of fibroblasts and endothelial cells, and lasts up to 3 weeks to completely repair the wounded area. The signature events during this phase include:

a) Fibroplasia, which consists in the formation of fibrous tissue to fill the wound area. During this phase, fibroblasts are stimulated by multiple cytokines and growth factors released by platelets, macrophages, keratinocytes, mast cells and endothelial cells. The fibroblasts become active, start to proliferate, and secrete MMPs and other proteases to allow cell migration through the provisional fibrin matrix. Active fibroblasts produce fibronectin, hyaluronic acid, proteoglycans, and collagen to form a new ECM that allows keratinocyte migration. The provisional fibrin matrix becomes the granulation tissue formed by a loose ECM, embedding blood vessels, macrophages, and fibroblasts. The fibroblasts differentiate in myofibroblasts, which contract to pull the wound together and decrease its area (Li and Wang, 2011; Darby et al., 2014). The myofibroblast differentiation process is mediated by TGFβ and CXCL8 and requires the interaction between fibronectin and αvβ5 integrin (Asano et al., 2006). Similarly, αvβ3 integrin upregulation contributes to establish an autocrine TGFβ loop in scleroderma fibroblasts (Asano et al., 2005). Interestingly, αvβ5 integrin is a reported Thy-1 ligand/receptor, and Thy-1 has been addressed as a negative regulator of latent TGFβ activation induced by fibroblast contraction, and as an inhibitor of myofibroblast differentiation through its interaction with αvβ5 integrin in lung cells (Zhou et al., 2010). TGFβ itself can also be regulated by mechanical strain. Within the wound, TGFβ is found inactive, and interacts with the latency-associated peptide (LAP) and the latent TGFβ binding proteins (LTBP). LTBPs (LTBP1, 3, and 4) link the TGFβ/LAP complex to the ECM, providing a dormant TGFβ pool that can be activated as wound healing progresses. The myofibroblasts express integrins that bind to the LAP, and mechanical force transduced by these integrins either from the ECM-bounded LTBP or from the myofibroblast contraction can release TGFβ by pulling LAP and allowing the TGFβ to bind its receptor (Munger et al., 1998; Annes et al., 2002; Annes et al., 2004; Wipff et al., 2007). The newly released TGFβ can increase the myofibroblast contractile phenotype and its ECM synthetic activity. Here, it is possible that Thy-1 in the same cell (*Cis* interaction) and through its RLD motif, compete with LAP and the ECM for integrin binding, maintaining the inactive state of the integrin and thus, allowing displacement of the other interactions without inducing myofibroblast differentiation (Zhou et al., 2010; Herrera-Molina et al., 2013). Blocking other integrins such as α3β1, α11β1, αvβ5 also inhibits myofibroblast development (Figure 1), highlighting the importance of integrin signaling during fibroplasia (Asano et al., 2006; Kim et al., 2009; Carracedo et al., 2010).

Thy-1 has a crucial role in suppressing proliferation and promoting differentiation of dermal fibroblasts. The lack of Thy-1 in dermal fibroblasts increases proliferation rates and reduces apoptosis by modulating β3 integrin function. Moreover, Thy-1^−/−^ fibroblasts display reduced expression of myofibroblast differentiation markers, such as αSMA, fibronectin, collagen I and III, and a reduced amount of biologically active TGFβ (Schmidt et al., 2015). Consistently, increased fibroblast proliferation in the absence of Thy-1 expression has also been described in fibrotic foci in lungs of patients with pulmonary fibrosis and fibroblasts derived from patients with hypersensitivity pneumonitis, supporting the idea that Thy-1 expression is necessary to avoid fibrosis (Ramirez et al., 2011). Alternatively, syndecan 4 regulates fibroblast migration during wound healing (Bass et al., 2011; Brooks et al., 2012). Interestingly, Thy-1-mediated migration and focal adhesion dynamics in embryonic fibroblasts depend on the recruitment of Partitioning-defective 3 (PAR3) to the cytoplasmic tail of syndecan 4. Consequently, PAR3 silencing inhibits FA disassembly triggered by Thy-1 stimulation, favoring fibroblast adhesion instead of migration (Valdivia et al., 2020). Further research is necessary to determine if the Thy-1/syndecan 4 interaction can also modulate fibroblast proliferation, differentiation, or ECM secretion.

b) Re-epithelialization starts approximately 16–24 h after injury, and during this phase, keratinocytes and MSCs work together to resurface the skin wound with new epithelium. Growth factors, chemokines, and cytokines released in the wound bed activate the keratinocytes adjacent to the wound edge to allow migration and proliferation (Peplow and Chatterjee, 2013). The keratinocyte-activated phenotype is characterized by changes in the cytoskeleton and membrane receptors, allowing keratinocytes to migrate. Wound keratinocytes express at least seven different integrins, which cooperatively control essential cell functions to promote proper re-epithelialization, including adhesion, migration, proliferation, survival, and basal membrane assembly (DiPersio et al., 2016). Migrating keratinocytes exhibit upregulation of keratins (K6, K16, and K17), MMPs, ECM proteins (Laminin), integrins (αvβ5, αvβ6, α5β1), and proteoglycans (perlacan and syndecans) (Figure 1) (Rousselle et al., 2019). In a porcine wound model, TGFβ stimulates expression of keratinocyte integrins during re-epithelialization of cutaneous wounds, and increases mRNA levels of integrin subunits α5, αv, and β5, with little effect on β1 in human keratinocytes (Gailit et al., 1994). Integrins and proteoglycans are essential for the keratinocytes to interact with the fibronectin-rich provisional matrix and later, with the collagen-rich matrix (Rousselle et al., 2019). Indeed, keratinocyte-specific β1 integrin knockout mice show a severe defect in wound healing. Furthermore, α3β1 integrin has been related to inhibition of migration and wound re-epithelialization in the skin, where α3-deficient keratinocytes migrate with an increased velocity and persistence (Margadant et al., 2009). Furthermore, the role of α6β4 integrin was determined in a human three-dimensional wound healing model by blocking β4 integrin with antibodies, which delay keratinocyte re-epithelialization (Egles et al., 2010). Additionally, epithelial-mesenchymal interactions between keratinocytes and fibroblasts generate a bidirectional signaling, in which keratinocytes stimulate fibroblasts to release growth factors that, in return, stimulate keratinocyte proliferation (Werner et al., 2007). Syndecan expression is increased in proliferating and migrating keratinocytes at the periphery of wounds in the skin (Figure 1) (Elenius et al., 1991). Particularly, syndecan 4 protein expression is significantly increased during tissue repair in the mouse and human dermis and is localized at the site of injury. Importantly, mice deficient in syndecan 4 experience delayed healing of excisional dermal wounds (Gallo et al., 1996; Echtermeyer et al., 2001). In a mouse wound healing model, Gallo and co-workers studied the upregulation of syndecan 4 throughout the dermis at the injury site; however, they observed that expression of syndecan 4 returns to basal levels in the re-epithelialization stage (Gallo et al., 1996). In addition, dermal fibroblasts isolated from syndecan 4 null mice exhibit decreased cell migration in wound healing assays *in vitro* and the inability to contract three-dimensional fibrin/fibronectin matrices during *in vitro* wound closure experiments (Midwood et al., 2004). On the other hand, Thy-1 expression in keratinocytes has been reported as a helpful marker of human keratinocyte stem cells (Nakamura et al., 2006). However, a role for Thy-1 in keratinocyte function and re-epithelialization has not been investigated yet.

The ECM plays a transcendental role not only during re-epithelialization, but also during every phase of the wound healing process (reviewed in Olczyk et al., 2014; Maquart, 2015; Xue and Jackson, 2015; Tracy et al., 2016; Rousselle et al., 2019). The secretion of ECM components occurs in a coordinated fashion in response to specific inflammatory and growth factors. The sequential deposition of ECM, along with cell-specific expression of receptors that interact with the ECM, are responsible for the synchronized recruitment of specific cell types to the wound area. Additionally, the ECM retains and modulates the delivery of growth factors and inflammatory molecules. Thus, aberrant ECM leads to delayed wound healing and abnormal scarring. Therefore, both ECM proteins and their integrin receptors play a key role in the re-epithelialization phase.

c) Angiogenesis is the formation of new capillaries from pre-existent blood vessels. This process starts approximately 4 days after the injury and forms the microvascular network throughout the granulation tissue. The granulation tissue is a stroma composed of connective tissue containing ECM proteins, as well as the cells necessary to allow the sprouting of blood vessels and subsequent wound closure. The reduction in blood supply and the exacerbated metabolism of the cells actively working in the healing process cause hypoxia, a major angiogenesis stimulus (Semenza, 2007; Castilla et al., 2012; Kimmel et al., 2016). This hypoxic environment induces an increase in the levels of the hypoxia inducible factor 1 (Hif-1) in macrophages, endothelial cells, fibroblasts, and keratinocytes (Andrikopoulou et al., 2011). Hif-1 activates the transcription of the main angiogenic factors, including VEGF, angiopoietin 1, TSP, and CXCL8. The presence of VEGF and macrophages induce the differentiation of endothelial cells into three different phenotypes: tip cells that secrete proteases, which degrade the basement membrane to allow them to detach from the vessel wall and migrate; proliferative stalk cells that elongate the new capillary sprout; and the quiescent phalanx cells that form the vessel lining. After the new capillaries are formed, PDGF, along with TGFβ and angiopoietin 1, stimulate mesenchymal cells to differentiate into pericytes and recruit other pericytes into the wound area. The pericytes wrap around immature capillaries and contribute to vessel maturation (Gaengel et al., 2009).

Even though the Thy-1 promoter lacks hypoxia regulatory elements, Thy-1 expression can be enhanced by Hif-1 downstream targets, such as cytokines and growth factors, as observed in the hypoxic-ischemic brain after injury or stroke. In a model of choroidal neovascularization, Thy-1 expression increases in primary endothelial cells after VEGF or CCL11 treatment, and after laser-induced injury. Moreover, Thy-1 ablation or inhibition reduces VEGF-induced migration and proliferation of endothelial cells, as well as VEGFR2, Rac and β3 integrin activation (Wang et al., 2016). Noteworthy, CCL11—a CCR3 ligand—is expressed by fibroblasts after the initial recruitment of lymphocytes during the inflammatory phase (Section 4). CCL11 positively affects neovascularization and enhances wound repair *in vitro* by inducing the migration of human primary dermal microvascular endothelial cells, dermal fibroblasts, and epidermal keratinocytes into the wound area (Salcedo et al., 2001; Park et al., 2017; Bunemann et al., 2018). Additionally, CCR3 is markedly expressed in dermal fibroblasts, and it is upregulated after cutaneous injury (Bunemann et al., 2018). Altogether, these findings suggest that CCL11 and its receptor CCR3 play a crucial role during the wound healing process; however, further studies are necessary to relate these effects to CCL11-CCR3-induced Thy-1 expression enhancement and to integrin/syndecan 4 signaling during wound closure.

Lee et al., showed that Thy-1 is expressed in newly formed vessels in four different models of adult angiogenesis: balloon injury of the carotid, tumor implantation in the cornea, renal artery ligation, and uterine capillaries formed during pregnancy (Lee et al., 1998). Accordingly, since embryonic angiogenesis occurs by different mechanisms than those of adult angiogenesis, Thy-1 is not detected on blood vessels of rat embryos. Moreover, cytokines such as IL-1β and TNFα upregulate the expression of Thy-1 in endothelial cells, and after carotid damage, Thy-1 appears in the newly formed vessels adjacent to zones with macrophage infiltration, indicating a biological function of Thy-1 during inflammation (Lee et al., 1998; Wen et al., 2013). Similarly, Thy-1 is upregulated in the EA.hy926 endothelial cell line when treated with TNFα (Brenet et al., 2020). Thy-1 expression in glioblastoma vasculature is associated with endothelial, smooth muscle, and pericyte cells, supporting Thy-1 function during angiogenesis (Inoue et al., 2016). Further *in vitro* studies have shown that Thy-1 overexpression decreases migration and tube formation of endothelial cells (Wen et al., 2013). Although the effect on tube formation can sound contradictory for a successful angiogenesis process, these results cannot be easily extrapolated to *in vivo* angiogenesis since they were performed in cells overexpressing Thy-1 and in Matrigel containing high amounts of cytokines and growth factors that can overstimulate the endothelial cells. One possible interpretation of these results is that the role of Thy-1 is to stabilize the newly formed blood vessels.

Most importantly, transcriptome analysis of blood vessels from chronic wound edge tissue showed that Thy-1 expression is 24-fold higher than in vessels from healthy human skin (Figure 1) (Roy et al., 2007). The biological significance of Thy-1 expression on the surface of endothelial cells after a skin wound is still not fully understood. However, Thy-1 would exert a role in recruiting lymphocytes and allowing trans-epithelial migration, as discussed in detail above (Section 4).

One of the essential factors for wound angiogenesis is αvβ3 integrin, a receptor for provisional matrix proteins, including vitronectin, fibronectin, and fibrin, which is expressed on the tips of angiogenic capillary sprouts (Figure 1) (Tonnesen et al., 2000). Blockage of αvβ3 integrin using antibodies or specific cyclic peptides inhibits the formation of granulation tissue and wound healing (Clark et al., 1996). Provisional matrix proteins, such as fibronectin and fibrin, increase αvβ3 integrin expression in endothelial cells, while a collagen-rich matrix has the opposite effect (Tonnesen et al., 2000). Noteworthy, the provisional matrix deposited during the hemostasis phase is subsequently replaced by collagen type I during the remodeling phase, suggesting that the distribution of αvβ3 at the tip of the capillaries is spatially and temporally regulated by the ECM. On the other hand, β1 integrins are expressed along the full length of the blood vessels, supporting the growing and sprouting of the vasculature when the new capillaries are extending (Yamamoto et al., 2015). β1 integrin expression increases when endothelial cells are plated on collagen-rich coated surfaces, suggesting a role in stabilizing the newly formed blood vessels at the end of the wound healing process (Tonnesen et al., 2000). Likewise, syndecan 4 is upregulated throughout the granulation tissue in endothelial cells and fibroblasts after injury (Gallo et al., 1996). *In vitro* assays using endothelial cells have shown that syndecan 4 is regulated by inflammatory factors, such as TNFα and IL-1β, and that decreasing syndecan 4 expression in endothelial cells impairs tube formation (Figure 1) (Vuong et al., 2015). Accordingly, mice lacking syndecan 4 expression showed reduced angiogenesis and delayed wound healing (Echtermeyer et al., 2001). The syndecan 4 cytoplasmic domain has also been described to be important for the function of FGF2, a potent angiogenesis inducer and signaling molecule in endothelial cells. Cells transfected with syndecan 4 mutated in the cytoplasmic domain show decreased PKCα activation, which is associated with impaired migration, proliferation, and tube formation (Horowitz et al., 2002). Full activation of FGF2 requires not only its interaction with the FGF receptor, but also its internalization (Goldfarb, 2001). Interestingly, FGF2 binds to and activates syndecan 4 (Simons and Horowitz, 2001). At the same time, active syndecan 4 leads to FGF2 complete activation by triggering FGF2 internalization through macropinocytosis in endothelial cells (Tkachenko et al., 2004). Recent reports indicate that FGF2 also upregulates syndecan 4 in endothelial cells at a high cell density, an event which might help repair damaged vascular endothelial cell layer or that could be related to the regulation of angiogenesis (Hara et al., 2020).

Thy-1 is also found in pericytes and mesenchymal progenitor cells that can differentiate into pericytes. A recent study identified two different pericyte populations associated with brain vessels, based on Thy-1 expression levels. Pericytes expressing high levels of Thy-1 exhibit a blunted inflammatory response, produce less ECM, and express lower levels of pericyte markers (e.g., SMA and PDGFR-β), when compared with pericytes expressing lower Thy-1 levels (Park et al., 2016). Although such heterogenicity in pericytes has not been determined after skin injury, these results suggest that Thy-1 may play a role in microvessel maturation and ECM deposition to form the granulation tissue (Figure 1). Moreover, pericytes expressing Thy-1 appear to secrete vesicles that exhibit Thy-1 on their surface and localize in the intercellular area (Bukovsky et al., 2001). Although these Thy-1 (+) vesicles are present between the epithelial-mesenchymal interphase, their biological role remains uncertain.

d) Peripheral nerve repair occurs by collateral reinnervation and nerve regeneration. After an injury, the undamaged axons are stimulated to produce collateral sprouting to reinnervate the skin. Severed nerves of the peripheral nervous system (PNS) can regrow the tips of two myelinated axon stumps and reconnect them to restore their homeostatic function in the skin (Gaudet et al., 2011). During this process, Schwann cells (SCs) present at the distal degenerating stump lose their myelin sheath and dedifferentiate to a progenitor-like cell to promote axonal regrowth. SCs leave the damaged nerve stump by directly interacting with fibroblasts accumulated at the wound bed, migrate, align, and form columnar bands of Büngner, which provide a substrate for axonal regeneration. Simultaneously, macrophages help to clean myelin and axon debris. The hypoxic environment and chemoattractant molecules released by SCs and other cell types, recruit macrophages/monocytes, which contribute to angiogenesis, as discussed above. Subsequently, SCs use the newly formed vasculature as a scaffold to guide the regrowing axons. Once the axons reinnervate, SCs differentiate and remyelinate the axons (Chernousov and Carey, 2000; Gaudet et al., 2011).

SCs express integrins that bind laminin (α2β1, α6β1, α6β4), collagen (α1β1, α2β1), and fibronectin (αvβ3, α5β1). Following a peripheral nerve lesion, both α5β1 and syndecan 4 levels are strongly increased in SCs nearby the wounded nerve (Lefcort et al., 1992; Milner et al., 1997; Chernousov and Carey, 2000; Goutebroze et al., 2003). On the other hand, fibroblasts in the wound area overexpress Thy-1 on their membranes. Since fibroblasts are responsible for guiding the SCs to the lesion site, this close interaction likely occurs by engagement of α5β1 integrin and syndecan 4 on the surface of SCs, with Thy-1 present at the fibroblast membrane (Figure 1). Similarly, our group has described in other glial cells (astrocytes) expressing αvβ3 and syndecan 4, that binding to Thy-1 promotes astrocyte adhesion to the ECM and subsequent cell migration (Hermosilla et al., 2008; Avalos et al., 2009; Kong et al., 2013; Valdivia et al., 2020).

An early investigation showed that Thy-1 expression in the PNS is restricted to the nodes of Ranvier within the sciatic nerve and that it colocalizes with laminin at the SC membranes (Liesi et al., 1990). Further research using transgenic rodents expressing fluorescent proteins under the control of the Thy-1 promoter (Thy-1-YFP) revealed that most of the neurons in the PNS were fluorescent, suggesting that Thy-1 is broadly expressed in the axons of adult peripheral neurons (Morris et al., 1983). Noteworthy, this kind of evidence is not functional and does not discard the possibility of a differential Thy-1 distribution in microdomains within the axon. Perhaps the area closer to the wound has a different density or less active Thy-1 than the rest of the axon, in order to favor the extension of the axon during repair. Indeed, experiments using the saphenous nerve crush model in Thy-1-YFP mice, followed by transcutaneous imaging to evaluate nerve generation, implied that the Thy-1 promoter is timely and spatially regulated after nerve damage. YFP expression driven by the Thy-1 promoter decreased nearby the crush injury. After 7 days, a second crush injury prompted a decrease in YFP levels distal to the crush site, up to complete disappearance 24 h later. After 2 days, YFP expression started increasing proximal to the crush site, to fully recover 7 days post-injury. Moreover, YFP expression at the crush site is exceptionally high compared with distal areas (Yan et al., 2011). This study suggests that the Thy-1 promoter is active in healthy and fully regenerated axons and at the site of nerve injury. Even though these cell-specific fluorescent reporters are highly popular to study peripheral nerve repair, very few studies have focused on the role of Thy-1 during this type of nerve repair in wounded skin (Feng et al., 2000; Pan et al., 2003; Kemp et al., 2013). For example, Thy-1 expressed on the axons of PNS facilitates the regeneration of peripheral nerves, such as the sciatic nerve (Kemp et al., 2013).

## 6 Remodeling Phase

The remodeling phase, also known as the maturation phase, is the final and longest phase of the wound healing process. It can last from 21 days to 2 years and occurs concurrently with granulation tissue formation. During remodeling, all the tissues formed in the previous phases, including the epidermis, vasculature, nerves, and myofibers, are mature and functional. In parallel, there is a decreased tissue cellularity due to apoptosis of fibroblasts, myofibroblasts, endothelial cells, pericytes, and inflammatory cells present within the granulation tissue. Additionally, the number of proteoglycans and glycosaminoglycans diminished, reducing the amount of water in the wound. The remodeling phase is characterized by wound contraction and ECM turnover. Myofibroblasts direct wound contraction, in which wound tensile strength increases as the collagen fibers are cross-linked by lysyl oxidase. The scar tissue has a tensile strength that is never greater than 80% of the unwounded skin, but this can be improved using MMP inhibitors. Throughout matrix turnover, MMPs released by fibroblasts and macrophages break down collagen type III and replace it with collagen type I, which is organized into parallel fibers (Toriseva and Kahari, 2009). As the remodeling process progresses, the ECM is reorganized and later suffers degradation, which is controlled by the balance of MMPs and tissue inhibitors of MMPs (TIMPs).

Remodeling is also regulated by growth factors such as TGFβ, PDGF, and FGF. TGFβ can increase collagen deposition by enhancing the synthesis of TIMPs. In contrast, PDGF and FGF increase the expression of collagenase. The balance between these growth factors controls the rate of remodeling. Importantly, PDGF also mediates fibroblast differentiation into myofibroblasts, which participate in wound contraction (Jinnin et al., 2005; Barrientos et al., 2008).

Macrophages are also crucial during remodeling since they adopt a fibrolytic phenotype to help engulf the ECM and cell debris. The removal of fibrous tissue is an important step during remodeling. When myofibroblasts express the CD47 on their surface (a “don’t-eat-me-signal”), they are not phagocyted by macrophages, resulting in excessive matrix deposition and hypertrophic scarring (Lech and Anders, 2013). As proposed, activation of the fibrolytic phenotype on macrophages and blockade of CD47 are relevant factors in reversing tissue fibrosis (Wernig et al., 2017). Interestingly, CD47, integrins, and syndecan 4 can bind and activate TSP1 and TSP2. Although controversial, TSP can regulate tissue remodeling and act as a negative regulator of wound healing (Kyriakides et al., 1999; Streit et al., 2000; Kyriakides et al., 2001; Agah et al., 2002; Kyriakides and Maclauchlan, 2009; Soto-Pantoja et al., 2014). Indeed, blocking of CD47/TSP signaling is effective in promoting wound closure and preventing necrosis of skin graft (Isenberg et al., 2008; Soto-Pantoja et al., 2014; Kale et al., 2021). However, the role of Thy-1/integrin/syndecan 4 trimolecular complex has not been studied in the context of CD47/TSP signaling in wounded skin.

Scar formation is the final step of wound repair. Occasionally, an imbalance may occur during ECM turnover, resulting in abnormal scar formation, such as hypertrophic or keloid scarring. Keloid scars are characterized and differentiated from hypertrophic scars by an excess of connective tissue forming a firm and raised scar that extends beyond the original wound limits and does not regress over time. A TGFβ signaling dysregulation, in which overexpression of TGFβ1 and β2, and blunted expression of β3, is believed to cause hyperactivation of fibroblasts, leading to increased ECM production. Consequently, collagen production is increased 3-fold in hypertrophic scars and 20-fold in keloids (Carswell and Borger, 2021). Likewise, a change in the expression of pro-inflammatory (IL-6 and IL-8) and anti-inflammatory (IL-10) cytokines have been associated with increased incidence of hypertrophic and keloid scarring (Berman et al., 2017). Despite this knowledge, the etiology of hypertrophic and keloid scarring is not fully understood yet; however, mechanical tension seems to be relevant, among other pathological factors (Butler et al., 2008). Physical tension has been proposed as a guide in keloid growth patterns, and skin areas subject to stretching have an increased incidence of keloid formation due to high mechanical stimulation (Akaishi et al., 2008; Carswell and Borger, 2021). Therefore, mechanical tension could play an important role during wound remodeling.

Every phase of the wound healing process is influenced by mechanical forces (Agha et al., 2011). Mechanical strain can alter the microenvironment of a healing wound, causing changes in cellular function, motility, and signaling (Kuehlmann et al., 2020). Fibroblasts are essential in supporting wound healing, and the signaling between the ECM-integrin-cytoskeleton is the classical pathway of mechanotransduction that regulates fibroblast viability, collagen production, and myofibroblast transformation during the remodeling phase (Bainbridge, 2013; Sun et al., 2016; Tschumperlin et al., 2018). Integrins are the main mechanotransducers of bidirectional information between cells and the ECM, and fibroblasts transduce mechanical strain predominantly through β1 integrins (Table 1) (Katsumi et al., 2004). Other important cells participating in wound healing that are affected by mechanical forces are keratinocytes, endothelial cells, and adipocytes (Fu et al., 2021). Cultured human keratinocytes and fibroblasts on a collagen membrane in a tensile device show asymmetric keratinocyte migration regulated by growth factors secreted by fibroblasts (Lu et al., 2016). Keratinocyte proliferation is regulated by mechanical strain through matrix-integrin signaling, epithelial-mesenchymal interactions, and Ca^2+^ channels, which transduce mechanical signals to PKC, PLC, and mitogen-activated protein (MAP) kinase pathways (Reichelt, 2007; Fu et al., 2021). Additionally, keratinocyte migration requires integrins to facilitate wound closure, as previously described during re-epithelialization (Section 5, c) (Kenny and Connelly, 2015). Indeed, β1 integrin can sense collagen and fibronectin ECM stiffness, and β1-null keratinocytes show a severe defect in skin wound healing (Figure 1) (Grose et al., 2002; Reynolds et al., 2008). Moreover, keratinocytes and fibroblasts rearrange their cytoskeleton in response to matrix stiffness, and the scar hardness and elevation observed in hypertrophic/keloid scarring can be influenced by rigidity-induced collagen production and cell proliferation (Hossain et al., 2005; Solon et al., 2007; Hadjipanayi et al., 2009). Focal adhesion kinase (FAK) is the most studied signaling pathway in skin mechanotransduction. FAK is activated downstream of integrins in fibroblasts and keratinocytes, and mechanical strain during wound healing leads to FAK hyperactivation, which correlates with hypertrophic scarring and fibrosis (Aarabi et al., 2007; Kuehlmann et al., 2020). In contrast, blunted FAK activation has been related to nonhealing wounds and delayed healing (e.g., diabetic wounds) (Wong et al., 2014; Liu W. et al., 2017). These data support the idea that the cell-centric view of wound remodeling is incomplete and suggests that tissue stiffness and mechanical strain within the wound may significantly influence the healing process. More investigations are necessary to unravel the mechanotransducer proteins that communicate internal with external forces in cells and their microenvironment.

The role of Thy-1 in integrin-mediated mechanotransduction has been mainly studied in *Trans* interactions, and little is known about *Cis* interactions (Hu and Barker, 2019). Thy-1, as well as integrins and syndecan 4, have been described as mechanotransducer proteins (Figure 1). Barker and co-workers showed that Thy-1 regulates fibroblast mechanotransduction. Thy-1 binding to αvβ3 integrin modulates rigidity-dependent Rho signaling, cytoskeleton remodeling, focal adhesion formation, and substrate rigidity sensing (Fiore et al., 2015). Furthermore, syndecan 4 is also considered a mechanotransducer protein (Bellin et al., 2009; Ross et al., 2013). Remarkably, integrins and syndecan 4 form a trimolecular complex with Thy-1, where two triads have been reported: α5β1/Thy-1/syndecan 4 in melanoma/endothelial cells and αvβ3/Thy-1/syndecan 4 in a neuron/astrocyte model (Fiore et al., 2014; Burgos-Bravo et al., 2020). However, further investigation is necessary to assess the specific role of Thy-1 and its binding partners during force transduction in cells involved in every phase of the skin wound healing process.

Additionally, tissue stiffness has been posited to drive stromal cells to differentiate into active myofibroblasts, which remodel the tissue, leading to fibrosis progression. Interestingly, fibroblasts show reduced Thy-1 expression in areas of active fibrogenesis in human idiopathic pulmonary fibrosis, and *in vitro* experiments have shown that the lack of Thy-1 expression in fibroblasts is enough to induce myofibroblast differentiation on soft matrix substrates (Hagood et al., 2005; Fiore et al., 2015; Fiore et al., 2018). Thus, Thy-1 expression in fibroblasts may represent a physiological mechanism relevant to fibrosis.

## 7 Mesenchymal Stem Cells

MSCs are multipotent stromal cells with the potential to differentiate into a variety of cell types, including adipocytes, chondrocytes, osteoblasts, myocytes, and stromal cells. MSCs can promote angiogenesis, granulation tissue formation, and epithelialization, which result in accelerated wound healing (Hu et al., 2018). MSCs have an anti-inflammatory effect during the inflammatory phase and can also stimulate fibroblasts, keratinocytes, and endothelial cells during the proliferative phase of wound healing.

Thy-1 (CD90) is a marker of MSCs and has a role regulating the fate of cells during cell differentiation. For instance, Thy-1 positive-MSCs follow osteogenic differentiation, while MSCs expressing reduced Thy-1 levels can promote adipogenic differentiation in cells isolated from dental pulp, adipose tissue, and amniotic fluid (Moraes et al., 2016; Picke et al., 2018). Additionally, strong evidence indicates that resident MSCs are activated to differentiate into other cell types, such as pericytes, myofibroblasts, and endothelial cells during wound healing and fibrosis (Yamaguchi et al., 2005; Wu et al., 2007; Sasaki et al., 2008; Lozito et al., 2009; Jeon et al., 2010; Nie et al., 2011; Ojeh and Navsaria, 2014; Park et al., 2016; Michelis et al., 2018). However, it is still unknown whether Thy-1 participates in MSC differentiation into these cell types in the skin. In addition, the mechanisms by which Thy-1 promotes MSCs differentiation are still controversial and need further investigation (Yang et al., 2020).

## 8 Current Therapeutic Strategies Targeting Thy-1, Integrins and Syndecan 4

Different therapeutic approaches to promote wound healing have been reported. Among these, ECM-based skin substitutes and cell-based therapies, such as implantation of MSCs, hydrocolloid pads, and chitosan dressing have shown promising results (Dreifke et al., 2015). In view of the important role that integrins, syndecan 4 and Thy-1 play in the wound healing process, we summarize and discuss here some of the current therapeutic efforts to modify these molecules and their interactions in order to improve wound healing outcomes and design potential future applications.

Integrins have been related with important roles along most of the wound healing stages (Figure 1). Impaired wound healing by overhealing (hypertrophic scar) or failure to heal (chronic wound) can result from aberrant integrin signaling. Many clinical studies in cancer and fibrosis have used integrin antagonists, such as blocking peptides, antibodies, and small molecules, to inhibit angiogenesis, cell migration, and proliferation (Silva et al., 2008; Lasinska and Mackiewicz, 2019; Su et al., 2020; Zhang et al., 2021). However, less is known about the advantages of these therapeutical approaches during wound healing of the skin.

To date, several pre-clinical studies in skin models have shown that integrin antagonists can potentially improve wound healing. For example, the antibody P1F6 (against αvβ5) and LM609 (against αvβ3) are effective in decreasing myofibroblast differentiation markers and TGFβ-induced gel contractility in human dermal fibroblasts (Lygoe et al., 2004). Similarly, both anti αvβ5 antibodies and RGD synthetic peptides have been used for blocking the interaction of this integrin with LAP-β1 and small latent complex (SLC) binding during the activation of latent TGFβ in dermal fibroblasts (Asano et al., 2006). Furthermore, blockade of other integrins, such as α3β1 and α11β1, also inhibits myofibroblast development (Kim et al., 2009; Carracedo et al., 2010). Thus, integrins related to TGFβ activation are biological targets for preventing fibrosis (Nishimura, 2009).

Integrins can also be targeted by creating substrates that mimic ECM components or binding peptides. The conjugation of integrin-targeting peptides to biomaterials has been shown transcendental for therapeutic success and has been recently reviewed (Zhao et al., 2020). In this context, for example, a thermoresponsive hydrogel formulated with laminin-derived peptides (PPCN-A5G81) that activate α3β1 and α6β1 integrins, has been tested in a diabetic mouse model, showing significant acceleration of wound closure (Zhu et al., 2018). During *in vitro* assays, the PPCN-A5G81 hydrogel induces cell spreading and migration of keratinocytes and dermal fibroblasts and controls the integrin α3- and α6-dependent proliferation of human dermal fibroblasts. As found in *in vivo* assays, the PPCN-A5G81 hydrogel accelerates the closure of excisional splinted wounds in diabetic mice by enhancing the granulation tissue formation and re-epithelialization (Zhu et al., 2018). Similarly, a peptide derived from angiopoietin 1 (QHREDGS), delivered as a soluble peptide, or collagen-chitosan hydrogel, or covering nanosheets of silk fibroin, positively influences re-epithelialization, angiogenesis, and granulation tissue formation to accelerate wound healing by binding αvβ3 and α5β1 integrins (Miklas et al., 2013; Xiao et al., 2016; Zhao et al., 2020). Moreover, a clinical trial indicated that the topical application of RGD peptide matrix scaffold, on partial-thickness scald burns in pediatric patients accelerates wound healing (Hansbrough et al., 1995). Therefore, to positively influence wound healing, the efforts to develop therapeutic tools can be focused on the activation or inhibition of specific integrins.

Some yet unexplored roles of syndecan 4 during wound healing include its function in regulating oligomerization and activation of chemokines, shedding from the cell surface in response to inflammatory molecules, and acting as a co-receptor for growth factors to enhance the signaling downstream of the growth factor receptor. In diabetic and obese mouse models, proteoliposomes containing syndecan 4 and PDGF-BB enhance PDGF-BB activity, improving wound healing. These proteoliposomes increase re-epithelialization and angiogenesis, compared to the treatment with PDGF-BB alone. The proportion of M1/M2 macrophages also improves, suggesting that the syndecan 4 proteoliposomes improve PDGF-BB efficacy in wound healing (Das et al., 2016). Most of syndecan 4 functions are mediated by their extracellular HS moiety (Bernfield et al., 1999; Tumova et al., 2000). Alternatively, heparanase, an endoglycosidase that cleaves HS from the cell surface and ECM, leads to the release and activation of HS-bound growth factors and cytokines that participate in the wound healing process. Heparanase itself is expressed in the granulation tissue and transgenic mice overexpressing heparanase show increased wound angiogenesis (Zcharia et al., 2005). Remarkably, topical application of heparanase enhances wound healing, vessel maturation and skin survival, suggesting that syndecan and other HS-proteoglycans can be important therapeutic targets (Zcharia et al., 2005; Fuster and Wang, 2010).

Thus far, the potential of Thy-1 as a therapeutic target has not been studied in skin wound healing. Some pre-clinical studies have provided some clues about the delivery of recombinant Thy-1 being beneficial in reversing fibrosis in biological systems other than skin. For instance, during idiopathic pulmonary fibrosis, fibroblasts lacking Thy-1 have increased proliferation and decreased myofibroblast differentiation markers. Similarly, Thy-1 deficit in an *in vivo* lung fibrosis mouse model is restored by administrating soluble Thy-1, showing that treatment with soluble Thy-1 therapeutically inhibits integrin-mediated fibrosis (Tan et al., 2019). Even though fibrosis mechanisms are considered to be similar between different tissues, the fibroblast heterogenicity within and between tissues suggest that these observations need to be carefully evaluated in skin wound healing fibrosis models.

## 9 Concluding Remarks

Here, we have reviewed diverse instances where either Thy-1, integrins or syndecan 4 are biologically relevant during wound healing. Specifically, we described that each of these proteins are upregulated during wound healing and that wound closure is delayed after decreasing the expression of any of these proteins. Our revision also pointed out to some putative functions of Thy-1, integrins and syndecan 4, based in observations performed in similar cell types in different biological systems. However, the effect of the Thy-1/integrin/syndecan 4 trimolecular complex remains mostly unexplored during skin injury.

Integrin or syndecan 4 modulation by itself has been shown to be effective in improving wound outcomes during pre-clinical assays. However, most of the successful treatments currently used in the clinic consist of complex skin substitutes or allographs composed of numerous ECM proteins, GAGs, growth factors, and cytokines, suggesting that a simplistic approach of modulating only one molecule may not be ideal.

Much less is known regarding the role of Thy-1 during skin wound and its potential as a therapeutic agent. Nonetheless, the fact that Thy-1 can act as the ligand of several integrins and syndecan 4, expressed by diverse cell types, highlights the importance of this glycoprotein as a broad modulator of cell function, and posits Thy-1 as an adequate biological target to develop future therapeutics for wound healing.

Wound healing is a complex process that involves an orchestrated plethora of events and requires the participation of many molecules and cells. An altered balance of these events results in aberrant wound phenotypes, where too little (chronic wounds) or too much (scarring and fibrosis) leads to unwanted outcomes. The trilogy of Thy-1/integrin/syndecan 4 proteins and its interaction as a trimolecular complex, either in *Cis* or *Trans*, offers an interesting window of opportunity for the development of new combined therapies to control the process of wound healing.

## Glossary

αSMA: α smooth muscle actin
bFGF: basic fibroblast growth factor
CD90: cluster of differentiation 90
ECM: extracellular matrix
FA: focal adhesion
FAK: focal adhesion kinase
Fas: TNF receptor superfamily, member 6
FasL: Fas ligand
FGF2: fibroblast growth factor 2
GAGs: glycosaminoglycans
GPCR: G-protein coupled receptor
HBD: heparin-binding domain
Hif-1: hypoxia inducible factor 1
IGF1: insulin growth factor 1
INFc: interferon c
LAP: latency-associated peptide
LPS: lipopolysaccharide
LTBP: latent TGF-β binding protein
MAPK: mitogen-activated protein kinase
MMPs: matrix metalloproteinases
MSCs: mesenchymal stem cells
NETs: neutrophil extracellular traps
NO: nitric oxide
PARP: poly ADP-ribose polymerase
PDGF: platelet-derived growth factor
PI-PLC: phosphoinositide-specific phospholipase C
PMA: phorbol 12-myristate 13-acetate
PMN: polymorphonuclear neutrophils
RAGE: receptor for advanced glycation end products
ROS: reactive oxygen species
SCs: Schwann cells
TCR: T-cell antigen receptor
TGFβ: transforming growth factor subunit β
Thy-1: thymus cell antigen 1
TIMPs: tissue inhibitors of matrix metalloproteinases
TLR: toll-like receptor
VEGF: vascular endothelial growth factor
YFP: yellow fluorescent protein
